# Cardiovascular and systemic determinants of exercise capacity in people with type 2 diabetes mellitus

**DOI:** 10.1177/2042018820980235

**Published:** 2021-01-27

**Authors:** Joanna M. Bilak, Gaurav S. Gulsin, Gerry P. McCann

**Affiliations:** Department of Cardiovascular Sciences, University of Leicester and The National Institute for Health Research (NIHR) Leicester Biomedical Research Centre, Leicester, UK; Department of Cardiovascular Sciences, University of Leicester and The National Institute for Health Research (NIHR) Leicester Biomedical Research Centre, Leicester, UK; Department of Cardiovascular Sciences, University of Leicester, Glenfield Hospital, Groby Road, Leicester LE39QP, UK

**Keywords:** diabetic cardiomyopathy, exercise capacity, heart failure

## Abstract

The global burden of heart failure (HF) is on the rise owing to an increasing incidence of lifestyle related diseases, predominantly type 2 diabetes mellitus (T2D). Diabetes is an independent risk factor for cardiovascular disease, and up to 75% of those with T2D develop HF in their lifetime. T2D leads to pathological alterations within the cardiovascular system, which can progress insidiously and asymptomatically in the absence of conventional risk factors. Reduced exercise tolerance is consistently reported, even in otherwise asymptomatic individuals with T2D, and is the first sign of a failing heart. Because aggressive modification of cardiovascular risk factors does not eliminate the risk of HF in T2D, it is likely that other factors play a role in the pathogenesis of HF. Early identification of individuals at risk of HF is advantageous, as it allows for modification of the reversible risk factors and early initiation of treatment with the aim of improving clinical outcomes. In this review, cardiac and extra-cardiac contributors to reduced exercise tolerance in people with T2D are explored.

## Introduction

The global prevalence of heart failure (HF) is growing at an alarming rate. In 2011, approximately 23 million people were living with HF worldwide, a figure that is expected to rise by 46% by 2030.^1^ In developed countries, up to one in five people are expected to develop HF during their lifetime.^2^ This is largely owing to the mounting pandemic of lifestyle-related diseases such as obesity and type 2 diabetes mellitus (T2D), which are intimately linked with each other, and with HF development.^3^ For example, United Kingdom estimates suggest that in the general population the prevalence of diabetes is 6%.^4^ However, in data from contemporary trials the prevalence of diabetes in HF patients ranges from 35% to 44%,^5,6^ with a particular predisposition towards HF with preserved ejection fraction (HFpEF). Furthermore, clinical outcomes for diabetes-associated HF are considerably worse for patients with T2D than those without,^7^ and development of HF in T2D is associated with the greatest risk of death and loss of lifespan than any other cardiovascular complication of T2D.^6^ HF is one of the most common complications of T2D, second only to peripheral vascular disease.^8^ Recognizing, preventing and treating HF in T2D is clearly a major priority for healthcare professionals and is considered a national priority in the United Kingdom.^9^

**Figure 1.**
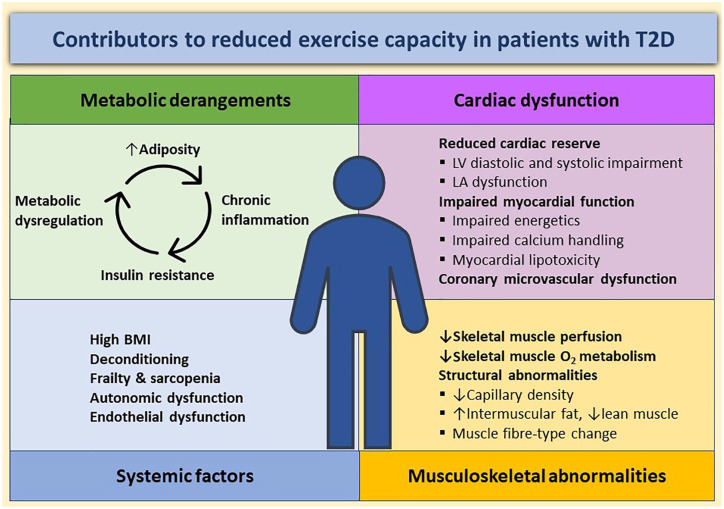
Contributors to reduced exercise capacity in T2D. Reduced exercise capacity in T2D is a net result of complex interactions between the biomechanics of obesity and frailty and the systemic and cardiovascular factors. Molecular mechanisms involved in the interactions between excess nutrients, adiposity, and chronic inflammation result in insulin resistance, which further propels the vicious cycle of metabolic dysregulation. Adapted from Del Buono MG, *et al.* J Am Coll Cardiol 2019;73(17):2209-25^19^ BMI, body mass index; LA, left atrium; LV, left ventricular; T2D, type 2 diabetes mellitus.

Despite not having overt signs or symptoms of HF or prevalent cardiovascular disease, numerous studies have reported a 20–30% reduction in peak oxygen consumption (VO_2peak_) in adults with T2D compared with controls.^10–13^ Exercise limitations occur early in the disease process and may be present in individuals with good glycaemic control^14^ and in those without clinically apparent cardiovascular disease.^14^ Importantly, these limitations in physical fitness correlate strongly with increased risk of cardiovascular and all-cause mortality, and HF.^15^ In combination, it is highly likely that early cardiovascular and systemic disturbances associated with T2D cause significant exercise limitation that predisposes to HF development. Enhanced understanding of the factors directly influencing exercise capacity in people with T2D may lead to the development of strategies to prevent or treat HF, as summarized in Figure 1. This review synthesizes the available evidence assessing predictors of exercise capacity in asymptomatic individuals with T2D. Links to mechanisms limiting exercise capacity specifically in HFpEF are explored. We emphasize the contributions of both cardiovascular and systemic factors that may lead to reduced physical fitness, highlighting areas of unmet research need and future strategies for targeted interventions.

## Physiological cardiovascular responses to exercise

In health, the cardiovascular system adapts and modifies its parameters in response to increased demand such as physical exertion, with the aim to facilitate tissue perfusion and oxygen delivery.^16^ At the microcirculatory level, perfusion pressure equates to mean arterial pressure which is regulated by interaction between cardiac output (a combination of heart rate and stroke volume) and systemic vascular resistance.^17^ During exercise, a positive chronotropic response and increased stroke volume (through re-direction of splanchnic and renal blood flow, increasing preload) result in increased cardiac output. The systemic vascular resistance in exercise-critical tissues such as the myocardium falls through local release of nitric oxide mediators and activity of cyclic guanosine monophosphate.^17^ The net result is a controlled change in mean arterial pressure, with improved perfusion of the exercise-critical muscle groups, such as the myocardium and skeletal muscle. The inability of one or more of these physiological parameters to adjust on exercise will result in failure of the cardiovascular system to meet tissue oxygen demands, leading to diminished exercise capacity.^17^

Cardiopulmonary exercise testing offers an assessment of individuals’ functional capacity through evaluation of gas exchange during exercise and thus an assessment of systems involved in both oxygen transport and utilization.^18^ Cardiopulmonary exercise testing allows for measurement of the volume of tissue oxygen uptake, which is a key parameter that offers insights into cardiac and pulmonary function, as expressed by Fick’s principle, according to which VO_2_ equates to cardiac output multiplied by the artero-venous gradient [C(a-v)O_2_].^18^ During ramp-like exercise, VO_2_ increases exponentially up to a steady state corresponding to peak exercise, and will adopt different patterns in patients with different aetiologies of HF.^18^ Any number of perturbations in T2D can interfere with the body’s normal physical responses to increased work, and thus affect the VO_2peak_.

## Systemic contributors to impaired exercise capacity

### Metabolic dysregulation and chronic inflammation in T2D

A number of theories have been proposed to explain the pathophysiology of the metabolic dysregulation, insulin resistance and development of endothelial dysfunction in adults with T2D and obesity.^20^ A chronic inflammatory state is induced by mitochondrial dysfunction and driven by chronic excess of nutrients, in particular the free fatty acids.^21–23^ Mitochondrial nutrient overload results in metabolic shifts towards generation of reactive oxygen species (ROS), which activate endothelial cytokine production, leading to direct endothelial damage and alterations of insulin signalling.^24^ This theory is based on the observation of higher and persistently elevated baseline levels of proinflammatory cytokines in obese individuals with T2D and insulin resistance as compared with lean controls.^25–31^ Furthermore, endothelial inflammation exerts pro-atherogenic effects, further compounding the cardiovascular risk in this cohort.^22,32^ In addition to promoting fatty streak deposition within the arterial wall, mitochondrial ROS results in reduced bioavailability of nitric oxide, which is essential to normal vascular homeostasis.^26^ The resultant impaired endothelial vasomotor mechanics are the hallmark of endothelial dysfunction and a key mechanism behind microvascular dysfunction, which is responsible for a range of the pathological sequelae of T2D, including left ventricular (LV) diastolic dysfunction.^32–36^ ROS toxicity leads to diastolic dysfunction by two mechanisms: first, ROS-mediated cardiomyocyte damage results in inflammation, apoptosis and fibrosis, directly contributing to LV diastolic dysfunction through remodeling,^22^ and second, ROS interact with endoplasmic reticulum, altering its structure and function primarily by altering the activity of the sarcoplasmic reticulum calcium pump, which is responsible for calcium sequestration during cardiomyocyte relaxation, thus leading to diastolic dysfunction.^37^ However, a recent systematic review of 11 studies did not show an association between exercise and reduced levels of inflammatory markers in adults with T2D.^38^

The volume and type of adipose tissue seems to have a significant effect on the propagation of the proinflammatory response. Brown adipose tissue and white adipose tissue play specific roles in energy metabolism and insulin homeostasis.^39^ Brown adipose tissue has an important role in regulating energy and glucose homeostasis, and has been associated with peripheral insulin resistance and glucose levels.^40^ Visceral white adipose tissue (around the trunk, upper body or abdomen) appears to be the major source of inflammatory markers in T2D, responsible for the production of inflammatory cytokines, thus contributing to the systemic inflammation and insulin resistance.^40^ Although insulin resistance has been associated with reduced VO_2peak_, this association has been described mainly from univariate analyses of small sample subjects with a risk of significantly overfitting the regression models.^41^ Although some older studies have shown association between glycaemic control (expressed as HbA1c) and exercise capacity,^42^ newer studies have not confirmed this association.^22^ Strict glycaemic control alone has not been shown to improve cardiovascular outcomes in patients with T2D.^42^

Changes within the systemic micro- and macro-vasculature play an important role in maintaining exercise capacity. In a study of 134 asymptomatic adults with T2D, reduced capillary blood flow to skeletal muscle was found and was positively correlated with VO_2peak_ independent of mean arterial pressure and cardiac output.^15^ This association was driven by capillary blood velocity reserve rather than capillary blood volume reserve, suggesting impaired endothelial vasomotive response to exercise.^13^ Furthermore, the association of capillary blood flow with VO_2peak_ was independent of mean arterial pressure, cardiac output reserve and other cardiac covariates, suggesting that pre-capillary factors, particularly endothelium-mediated vasodilation, may be responsible.^13^ In another study, which compared 20 uncomplicated T2Ds with 20 T2Ds with microvascular complications, the latter group had abnormal skeletal muscle capillary responses to periodic contractile exercise,^43^ thus reflecting an underlying abnormality in microvascular recruitment.^20^ These findings again implicate endothelium-mediated vasodilation, which is responsible for exercise hyperaemia and known to be impaired in diabetes.^17^ Insulin increases limb blood flow in a dose dependent fashion; however, this mechanism is ineffective in the presence of insulin resistance and is compounded by reduced local availability of nitric oxide, as present in microvascular dysfunction.^11^

### Clinical predictors

A number of studies evaluating determinants of exercise capacity have linked clinical characteristics to exercise capacity (Table 1). The strongest independent predictors of VO_2peak_ have been age and sex. In the largest to date study of over 5000 participants with T2D, peak exercise capacity was higher for males compared with females and there was a consistent 5–10% reduction in metabolic equivalents of tasks (METs) per decade of life.^44,45^ Body habitus [both increased waist circumference and body mass index (BMI) ⩾30 kg/m^2^] were also independently associated with reduced exercise tolerance (all *p* < 0.001).^46^ In addition, duration and severity of T2D (expressed as insulin resistance) have been linked to reduced VO_2peak_ in adults with T2D.^45^ However, it is important to note that these associations have been produced from univariate analysis of studies often involving small sample subjects, reported in subjects with T2D regardless of disease severity, mode of assessment of exercise capacity or presence of LV dysfunction, which confounds the findings.

**Table 1.** Clinical determinants of exercise capacity in T2D.

Author	T2D group(s)	Inclusion/exclusion criteria	Method of assessment	Predictors of ↓ VO_2peak_	Comments
Gulsin *et al.*^47^	*N* = 247 T2D age 51.8 ± 11.9 years, 55% males, HbA1c 7.4 ± 1.1%	Incl.: T2D, asymptomatic	CMR for MPR	Myocardial perfusion reserve (*β* = 0.822, *p* = 0.006) and E/e′ (*β* = -0.388, *p* = 0.001) were independently associated with VO_2peak_ in subjects with T2D	In subjects with T2D, significant correlations were observed between VO_2peak_ and age, T2D duration, BP, absolute and indexed LV volumes, LV EF, LV mass, LV GLS, average E/e′ and MPR
		Excl.: T1D, IHD, HF, CKD	TTE for E/e′		
			CPET for VO_2peak_		
Vukomanovic *et al.*^48^	*N* = 70 T2D, uncomplicated, age 52 ± 7 years, 56% M, HbA1c 7.5 ± 1.2 controls	Incl.: T2D	CPET for VO_2peak_	GLS (–21.6 ± 2.8 *versus* −18.4 ± 2.3%, *p* < 0.001) and circumferential strain (−22.0 ± 2.9 *versus* −19.5 ± 2.6%, *p* < 0.001) were reduced in DM *versus* controls. VO_2peak_ significantly lower in T2D (27.0 ± 4.3 *versus* 20.7 ± 4.0 mL/kg per min, *p* < 0.001)	Age and sex were not forced into the regression model. Furthermore, subjects were combined with controls in the regression model
		Excl.: HTN, HF, CAD	TTE for GLS and GCS		
Kosmala *et al.*^49^	*N* = 510 (292 T2D)	Incl.: asymptomatic patients with T2D, HTN and BMI ⩾30 kg/m^2^	Diastolic dysfunction (E/e′ >13) or strain >18%	↓VO_2peak_ associated with LV strain and presence of LVH (*p* < 0.001)	↓VO_2peak_ correlated with ↑components of abnormal values; however, not adjusted for age or sex
	Stage AHF: T2D (*n =* 186, 70%), HbA1c 7 ± 1.6%, log HOMA-IR 0.41 ± 0.36				
			CPET for VO_2peak_	Multivariate: presence of stage B HF independently associated with ↓VO_2_ peak. Stage B HF (>1 imaging variable present) associated with lower VO_2peak_ (beta ¼ 0.20; *p* < 0.0001) and METs (beta ¼ 0.21; *p* < 0.0001	
	Stage BHF: T2D (*n =* 106, 44%) HbA1c (7 ± 1.4%), log HOMA-IR 0.47 ± 0.27				
		Excl.: complications of T2D, IHD			
Fang *et al.*^44^	*N* = 170, 53% M, age 56 ± 10 years	Incl.: T2D	Abnormal EC defined as: METs = 18 × 0.15 × age	Univariate: ↓EC associated with ↓diastolic function.↑ EC: males (*r* = 0.26, *p* < 0.001), preserved Em (*r* = 0.43, *p* < 0.001), and preserved HRR (*r* = 0.42, *p* < 0.001)	NS correlation in univariate analysis between METs, LV EF, insulin or cardiovascular drugs
		Excl.: LV EF <50%, IHD			
			Abnormal HRR: ⩽18 beats.min^−1^, echo: strain, Em		

AHF, stage A heart failure; BHF, stage B heart failure; BMI, body mass index; BP, blood pressure; CAD, coronary artery disease; CMR, cardiac magnetic resonance; CPET, cardiopulmonary exercise test; DM, diabetes mellitus; EC, exercise capacity; E/E’, ratio of early mitral inflow velocity and mitral annular early diastolic velocity; EF, ejection fraction; Em, early mitral inflow velocity; GCS, global circumferential strain; GLS, global longitudinal strain; HF, heart failure; HOMA-IR, homeostatic model assessment of insulin resistance; HRR, heart rate recovery; HTN, hypertension; IHD, ischaemic heart disease; LV, left ventricle; LVH, left ventricular hypertrophy; M, male; MET, metabolic equivalent of task; MPR, myocardial perfusion reserve; T2D, type 2 diabetes; TTE, transthoracic echocardiography; VO_2peak_, peak oxygen consumption.

### Skeletal muscles and anaerobic metabolism

In T2D, microangiopathy contributes to skeletal muscle dysfunction though impaired perfusion and oxygen extraction during exercise. In addition to impaired exercise hyperaemia, skeletal muscle oxygen extraction is impaired in adults with T2D compared with non-diabetic individuals of similar anthropometric features and equally sedentary lifestyle.^16^ During graded exercise at 60%, 70% and 100% VO_2max_, those with T2D achieved significantly lower workloads than controls.^50^ This corresponded to a minimal rise in stroke volume, and no change in cardiac output between 60% and 100% VO_2max_ despite adequate rise in heart rate. Furthermore, VO_2max_ was correlated with a-vˉ O_2_ difference (19% lower in T2D, *p* < 0.001) but not with cardiac output, suggesting that impaired maximal total body O_2_ extraction contributed to lower VO_2max_ in T2D patients.^51^ This may be in part explained by presence of diastolic dysfunction; however, the study did not include echocardiographic data. Diabetes-mediated endothelial dysfunction results in reduced local availability of nitric oxide, which is the key mediator of exercise induced hyperaemia within the skeletal muscles.^15^ The lower a-vˉ O_2_ difference may suggest that T2Ds had impaired peripheral oxygen extraction and were more reliant on anaerobic metabolism. Impaired VO_2max_ in T2D may therefore be related to poor peripheral oxygen extraction and reliance of anaerobic metabolism,^15^ which would be explained by presence of endothelial dysfunction.

### Autonomic dysfunction

Systemically, diabetes-mediated cardiac autonomic dysregulation results in impaired chronotropic response to exercise and in turn lowers myocardial ability to modulate cardiac output through heart rate.^52^ This confers reduced exercise tolerance through the inability of cardiac output to meet the metabolic demands of tissues. Several studies have reported impaired heart rate response and heart rate recovery in T2D, with positive associations with VO_2peak_ on univariate analysis^14,44,45,51^ but not in any multivariable analysis. Nevertheless, autonomic dysregulation is one of the many factors contributing to impaired exercise tolerance in T2D.

### Frailty

Increasingly, T2D is being recognized as a driver of accelerated metabolic ageing and physical deconditioning, which manifest as a state of physical frailty.^53^ People with T2D are up to five times more likely to suffer from frailty than individuals without diabetes.^54^ The diabetes-related frailty phenotype is now regarded as a major contributor to low physical functioning in people with T2D.^54^ It is, however, distinct from the traditional frailty phenotype that is prevalent in elderly, low body weight people. Rather, frailty in T2D occurs in younger as a well as older age groups, is related to obesity in the presence of sarcopenia, but still manifests as low physical fitness and reduced quality of life.^54^ To our knowledge, however, no studies have directly evaluated the contribution of frailty in T2D to objective measures of aerobic exercise capacity. Given the considerable impact that frailty will have on exercise capacity, identifying and treating frailty in T2D is an area that warrants further study.

## Cardiovascular contributors to impaired exercise capacity

### The pathophysiological mechanisms leading to development of diabetic cardiomyopathy

**Figure 2.**
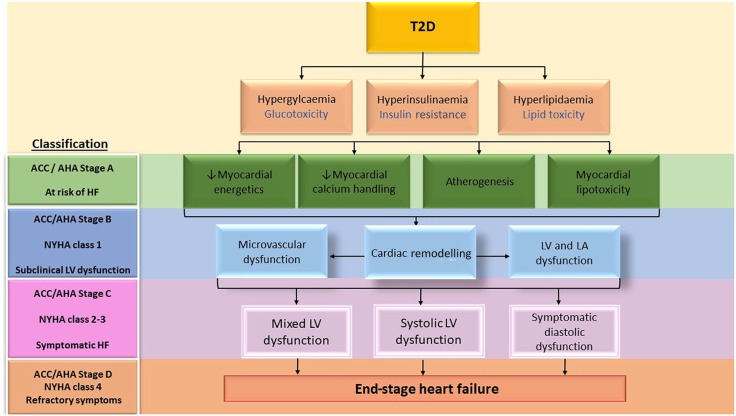
Pathological alterations leading to diabetic cardiomyopathy in relation to stages of progression of heart failure. ACC/AHA, American College of Cardiology/American Heart Association; HF, heart failure; LA, left atrium; LV, left ventricular; NYHA, New York Heart Association; T2D, type 2 diabetes mellitus.

The pathological myocardial alterations characteristic of diabetic cardiomyopathy begin early in the course of T2D and are present in otherwise asymptomatic individuals, suggesting a latent phase of cardiovascular dysfunction.^3^ Cardiac dysfunction in diabetes is thought to lie on a continuum ranging from asymptomatic diastolic dysfunction though subclinical systolic dysfunction and then overt HF.^55^ Reduction in exercise tolerance is amongst the first marker of stage B HF (defined as structural or functional LV alterations in the absence of symptoms) in diabetic cardiomyopathy and a 10% reduction in an individual’s exercise tolerance in the presence of detectable cardiomyopathic changes would automatically class them as stage-II HF by the New York Heart Association (NYHA).^56^ Figure 2 summarizes the stages of progression of diabetic cardiomyopathy, based on the presence of cardiomyopathic changes and symptomatology, with reference to the American College of Cardiology/American Heart Association (ACC/AHA) and NYHA HF classification scores, and Table 2 summarizes cardiac predictors of reduced exercise capacity.

**Table 2.** Cardiac predictors of exercise capacity in T2D.

Author	T2D group(s)	Inclusion/exclusion criteria	Method of assessment	Predictors of ↓ VO_2_peak	Comments
Gulsin *et al.*^47^	*N* = 247 T2D age 51.8 ± 11.9 years, 55% M, HbA1c 7.4 ± 1.1%	Incl.: T2D, asymptomatic	CMR for MPR	MPR (β = 0.822, *p* = 0.006) and E/e′ (β = –0.388, *p* = 0.001) were independently associated with VO_2peak_ in subjects with T2D	In subjects with T2D, significant correlations were observed between VO_2peak_ and age, T2D duration, BP, absolute and indexed LV volumes, LV EF, LV mass, LV GLS, average E/e′ and MPR
		Excl.: T1D, IHD, HF, CKD	TTE for E/e′		
			CPET for VO_2peak_		
Vukomanovic *et al.*^48^	*N* = 70 T2D, uncomplicated, age 52 ± 7 years, 56% M, HbA1c 7.5 ± 1.2 Controls	Incl.: T2D	CPET for VO_2peak_	GLS (−21.6 ± 2.8 *versus* -18.4 ± 2.3%, *p* < 0.001) and circumferential strain (−22.0 ± 2.9 *versus* −19.5 ± 2.6%, *p* < 0.001) were reduced in T2D *versus* controls. VO_2peak_ significantly lower in T2D (27.0 ± 4.3 *versus* 20.7 ± 4.0 mL/kg per min, *p* < 0.001).	Age and sex were not forced into the regression model. Furthermore, regression was calculated combined with controls
		Excl.: HTN, HF, CAD	Echo for GLS and GCS		
Kosmala *et al.*^49^	*N* = 510 (292 T2D)	Incl.: asymptomatic patients with T2D, HTN and BMI ⩾30 kg/m^2^	Diastolic dysfunction (E/e′ >13) or strain >18%	↓VO_2peak_ associated with LV strain and presence of LVH (*p* < 0.001),	↓VO_2peak_ correlated with ↑components of abnormal values; however, not adjusted for age or sex
	Stage A HF: T2D (*n* = 186, 70%), HbA1c 7 ± 1.6%, log HOMA-IR 0.41 ± 0.36				
				Multivariate: presence of stage B HF independently associated with ↓VO_2peak_. Stage B HF (>1 imaging variable present) associated with lower VO_2peak_ (beta ¼ 0.20; *p* < 0.0001) and METs (beta ¼ 0.21; *p* < 0.0001	
		Excl.: complications of T2D, IHD	CPET for VO_2peak_		
	Stage B HF: T2D (*N* = 106, 44%) HbA1c (7 ± 1.4%), log HOMA-IR 0.47 ± 0.27				
Fang *et al.*^44^	*N* = 170, 53% M, age 56 ± 10 years	Incl.: T2D	Abnormal EC defined as: METs = 18 × 0.15 × age Abnormal HRR: ⩽18 beats.min^−1^, echo: strain, Em	Univariate: ↓EC associated with ↓diastolic function.↑ EC: males (*r* = 0.26, *p* < 0.001), preserved Em (*r* = 0.43, *p* < 0.001) and preserved HRR (*r* = 0.42, *p* < 0.001).	NS correlation in univariate analysis between METs, LV EF, insulin or cardiovascular drugs
		**Excl.:** LV EF <50%, IHD			

BMI, body mass index; BP, blood pressure; CAD, coronary artery disease; CKD, chronic kidney disease; CMR, cardiac magnetic resonance; CPET, cardiopulmonary exercise test; EC, exercise capacity; E/E′, ratio of early mitral inflow velocity and mitral annular early diastolic velocity; EF, ejection fraction; Em, early mitral inflow velocity; GCS, global circumferential strain; GLS, global longitudinal strain; HF, heart failure; HOMA-IR, homeostatic model assessment of insulin resistance; HRR, heart rate recovery; HTN, hypertension; IHD, ischaemic heart disease; LV, left ventricle; LVH, left ventricular hypertrophy; M, male; MET, metabolic equivalent of task; MPR, myocardial perfusion reserve; T1D, type 1 diabetes; T2D, type 2 diabetes; TTE, transthoracic echocardiography; VO_2peak_, peak oxygen consumption.

The pathogenesis of diabetic cardiomyopathy is complex and incompletely understood.^3^ Myocardial steatosis, altered myocardial energetics, and impaired calcium handling have all been implicated. *In vivo* studies have confirmed elevated myocardial triglyceride content in T2D^57,58^ and myocardial steatosis has been linked to both diastolic and systolic strain,^59^ linking steatosis with development of cardiac dysfunction. Myocardial energy metabolism (as assessed by the myocardial Creatinine phosphate/ATP ratio) is reduced in T2D and this is exacerbated by exercise.^60^ Abnormalities of myocardial calcium handling *via* impairments in the sarcoplasmic reticulum Ca^2+^ ATPase (SERCA) have been implicated in HF, but not in T2D. SERCA2a activity declines in late stage HF^61^ and decreased levels of SERCA2a have been found in cardiac tissues isolated from humans and animals with HF.^62^ Importantly, low SERCA2 levels have been correlated to poor clinical outcomes.^62,63^ Therapeutic approaches aiming to boost the myocardial SERCA2a levels have produced disappointing results.^61,64^ No studies, however, have directly evaluated links between myocardial steatosis, calcium handling, or energetics and impaired aerobic exercise capacity.

The predominant HF phenotype in people with T2D is HFpEF, which accounts for up to 83% of newly diagnosed cases of HF.^65^ In two contemporary large-scale HF trials of angiotensin-neprilysin inhibitor sacubitril-valsartan – PARAGON-HF^52^ and PARADIGM-HF^5^ – prevalence of diabetes was 44% and 35%, respectively, and in our own HFpEF cohort 54% of 140 patients had T2D.^66^ People with T2D appear to be particularly prone to development of diastolic LV impairment, although systolic LV impairment often co-exists. A significant proportion of patients with diastolic impairment are asymptomatic, which poses a clinical challenge as diastolic dysfunction even in isolation is associated with poor outcomes.^3,67^ Over the past three decades, the rapid evolution of advanced non-invasive cardiac imaging techniques has enabled detailed evaluation of cardiovascular structure and function *in vivo.* Application of these techniques has provided key insights to the relationship between cardiovascular function and exercise capacity in T2D, shedding light on early perturbations that may lead to HF (Table 2).

### LV diastolic dysfunction

LV diastolic dysfunction is widely regarded as the earliest functional change occurring in diabetic cardiomyopathy.^3^ The reported prevalence of LV diastolic dysfunction in asymptomatic subjects ranges between 15% and 78%^3,66,68^ and differs according to imaging technique used. Subclinical diastolic dysfunction is frequently observed in asymptomatic, sedentary T2D even in the absence of microvascular complications, and is associated with impaired exercise tolerance (time and METs achieved).^10,50^ Several inverse correlations between indices of impaired LV relaxation and VO_2peak_ in asymptomatic individuals have been identified, including smaller cardiac size (LV end-diastolic volume, *r* = 0.67)^45^ and attenuated increase in stroke volume during exercise,^14^ suggesting that impaired LV compliance may herald development of diastolic dysfunction. Invasive measurements of pulmonary capillary wedge pressure offer further insight on the impaired LV diastology in diabetes.^50^ The VO_2peak_ and peak cardiac output were lower in T2D than in controls, and the pulmonary capillary wedge pressure rose significantly more during exercise in T2D than in controls (148% *versus* 109% increase at peak exercise, *p* < 0.01).^50^ However, the numbers included in the study were small and limited to females only, which precludes generalization to the whole population and conclusions on the causative associations of reduced exercise tolerance.

The pathological myocardial and systemic changes precede development of overt diabetic cardiomyopathy, and can exist even in the absence of symptoms. The clinical importance of these findings is recognized by the ACC/AHA, who classify this as stage B HF (SBHF).

### LV systolic dysfunction

Despite the association of T2D with HF, few studies have shown that diabetes causes a reduction in LV ejection fraction (EF), which remains the most utilized form of assessing LV performance. Furthermore, the evidence to suggest a relationship between VO_2peak_ and systolic LV EF is lacking.^69,70^ Subclinical LV dysfunction, as measured by impaired myocardial strain and strain rates, is increasingly reported in T2D, and affects all layers of myocardium, from apex to base.^60,71^ Individuals with T2D have reduced global longitudinal strain (GLS) rate compared with controls, and it is detectable with a range of imaging techniques, including speckle tracking echocardiography^69^ and cardiac magnetic resonance (CMR) feature tracking.^72^ These impairments with GLS worsen over time,^73^ inversely correlate with indices of glycaemic control^48^ and have been found to be an independent predictor of cardiovascular events in longitudinal studies.^73^ GLS may thus offer an incremental prognostic value in this cohort, especially as GLS has been shown to be superior to LV EF at identifying patients with reduced exercise capacity.^74^ Several small observational studies have shown that GLS and global circumferential strain (GCS) may be independently associated with VO_2peak_. In a 100 patient study of adults with T2D, a GLS value of −17.3% had excellent sensitivity of 0.89 [95% confidence interval (CI) 0.79–0.95] and specificity of 0.91 (95% CI 0.71–0.99) to identify patients with a VO_2peak_ of <20 mL/kg per min independent of age and sex.^70^

In another study of 80 asymptomatic T2D, GLS (−21.6 ± 2.8 *versus* −18.4 ± 2.3%, *p* < 0.001) and GCS (−22.0 ± 2.9 *versus* −19.5 ± 2.6%, *p* < 0.001) were significantly reduced in all myocardial layers in T2D patients^73^ and were associated with lower VO_2peak_ independently of other clinical and echocardiographic parameters of LV structure, and systolic and diastolic function.^73^

### Coronary microvascular dysfunction

Several studies have shown reduced myocardial perfusion reserve (MPR) in T2D, which is now being recognized as part of the pathophysiology of HF in T2D as well as HFpEF.^35,60,71,75,76^ Our group has assessed the association between aerobic capacity and cardiac structure and function in asymptomatic T2D, using a combination of multiparametric CMR and echocardiography (see Table 2).^47^ Even after exclusion of subjects with reversible perfusion defects, the overall MPR in the diabetic cohort was lower than in matched controls (2.60 ± 1.24 *versus* 3.54 ± 1.15, respectively, *p* < 0.001). On both univariate and multivariable analysis in subjects with T2D, the ratio of early mitral inflow velocity and mitral annular early diastolic velocity (E/e′) (β = −0.388, *p* < 0.001) and MPR (β = 0.0822, *p* = 0.006) were significantly associated with VO_2peak_ independent of age, sex, ethnicity, smoking status and systolic blood pressure.^47^ This may be explained by the fact that myocardial perfusion must increase incrementally during exercise to meet the metabolic demands of tissues.^77^ A similar relationship has been documented in patients with severe aortic stenosis.^77^

### Left atrial dysfunction

Left atrial (LA) enlargement is increasingly recognized for its association with adverse cardiac outcomes, including atrial fibrillation, stroke and heart failure.^78^

In addition, the LA plays an important role in cardiovascular response to exercise, specifically if LV diastology is also impaired. In diastolic LV dysfunction, prolonged relaxation time leads to a greater dependence on the atrial contribution at end-diastole for optimal filling.^79^ Diastolic filling time is inversely proportional to heart rate and this association is more pronounced during exercise. Reduced LV relaxation time leads to a greater dependence on atrial contribution at end-diastole for optimal filling.^74^ Impaired atrial systolic function will compromise cardiac output with effort, which highlights the role of the LA for maintaining exercise capacity.^80^

Atrial geometry, function and electrophysiological alterations are well-defined in patients with HFpEF and in atrial fibrillation, and have been closely linked with reduced exercise capacity and HF-related outcomes.^81–92^ However, atrial myopathy in T2D appears distinct. In adults with T2D, abnormalities of LA geometry have been described but the results are contradictory. While some studies reported smaller LA volumes in subjects with T2D^89^ others have shown the opposite.^78^ Smaller atrial volumes are observed in T2D in the presence of HFpEF, a disease typically associated with increased LA volumes.^86,91,92^ We have recently compared patients with HFpEF with and without T2D.^66^ Despite higher BMI and higher filling pressures (E/e′) than the patients without T2D, the diabetic HFpEFs had smaller LA volumes, suggesting that atrial myopathy in T2D is different from LA dilatation observed in HFpEF.^90^ T2D has been proposed as an independent risk factor for LA impairment, regardless of co-existent hypertension or presence of LV diastolic dysfunction.^89,90^ Whilst the link between LA dysfunction and reduced exercise capacity is well established in HFpEF, there is paucity of data in asymptomatic people with T2D. Abnormalities of LA function are utilized as prognostic markers in heterogenous cohorts of HFpEF patients which included T2D: increased indexed LA volume (>32 mL/m^2^)^87,90^ and reduced LA peak strain,^93–96^ reservoir, conduit and pump function^66^ have all been found to independently correlate with an increased risk of major adverse cardiovascular events^50,68,86^ and hospitalization for HF.^85^

## Strategies to improve exercise capacity

### Weight loss and exercise

Weight loss confers a number of clinically important benefits in patients with T2D.

Weight loss achieved through bariatric surgery^97,98^ or low-calorie meal replacement diet^58^ results in remission to a non-diabetic state, with a strong correlation between the extent of weight loss and reversal of T2D. However, the same effects are not seen in more advanced T2D (defined by insulin therapy) or with longer disease duration.^58,97,98^ Sustained weight loss also confers direct beneficial cardiovascular effects in obese adults without T2D, with reductions in LV mass, volumes, arterial stiffness and diastolic function as measured by CMR.^3^ Improved diastolic function, energetics and reduced myocardial triglyceride content, which may confer benefit to exercise tolerance, have been reported in obese individuals following bariatric surgery-mediated weight loss.^99^ Importantly, bariatric surgery can achieve sustained weight loss (in up to one-fifth of patients), sustained remission of diabetes (in up to one-third of patients) and lower rates of major adverse cardiovascular events (including HF) in people with T2D and obesity.

In addition to benefits on cardiac function, weight loss simply improves physical function in adults with T2D by reducing the biomechanical burden of moving around.^47^ However, the cardiovascular benefits of weight loss alone do not directly translate to significant improvements in objective measures of aerobic exercise capacity. In fact, a number of studies have reported reduced strength and VO_2max_ in individuals exposed to caloric restriction alone.^47^ In a study of 52 obese individuals, daily calorific reduction of 20% mediated weight loss of approximately 7% of body mass over a 12 week period and corresponded to an approximately 6% reduction in absolute VO_2max_, an effect that was attenuated by exercise.^99–101^ Conversely, exercise alone resulted in 15% improvement in the VO_2max_, even in the absence of weight loss. The combination of modest exercise (4.4 ± 0.5 h/week) and 20% calorific reduction attenuated the reduction in lean mass and aerobic capacity that occurred with calorific reduction alone. Weight loss achieved through exercise confers the greatest benefits on preservation of lean mass and increase in VO_2max_, but the required amount of exercise to cause weight loss is substantial (7.4 ± 0.5 h/week) and may be challenging to achieve in deconditioned, overweight individuals in the real world.^100^

Our group has also assessed the impact of lifestyle interventions on cardiac function and exercise capacity in younger obese adults with T2D.^102^ We undertook a 12-week randomized trial comparing a supervised exercise programme or low energy meal replacement diet. A significant improvement in the primary outcome of diastolic function was observed in the exercise arm, despite only small reductions in weight, BMI and exercise capacity. By contrast, in the diet arm there was dramatic overall weight loss (median 13.6 kg and fall in BMI of 4.8 kg/m^2^) accompanied by a mean HbA1c decrease of 0.75%, with 83% of participants achieving T2D remission. However, only a small increase in VO_2peak_ when corrected for body weight (1.9 mL/kg per min) was observed, but there was no change in absolute VO_2peak_.^103^ There were no significant improvements in myocardial perfusion or remodelling with exercise.^103^ Although exercise has been found to improve endothelial function, it is possible that the small sample size and short duration (12 weeks) of follow-up precluded these effects from fully manifesting in this study. The lack of improvement in VO_2peak_ may be explained by loss of lean tissue mass.^103^ Even in obese individuals, weight loss resulting from calorific restriction results in loss of lean tissue as fat free mass in a 1:4 ratio with adipose tissue. The predominant site of reduction in lean body mass is the skeletal muscle, which when coupled with possible reductions in the functional capacity of the musculature with weight loss, limit the magnitude of benefits realized.^103^

### Pharmacological treatments

In addition to improving diabetic control, several antidiabetic treatments have been shown to have cardioprotective effects, but data on their efficacy in improving anaerobic capacity are sparse. Two large randomized controlled trials – LEADER (liraglutide)^104^ and PIONEER-6 (semaglutide)^105,106^ – have shown a reduction on atherosclerotic cardiovascular events with glucagon-like receptor 1 agonist treatment, and in the case of semaglutide a nearly 14% weight loss.^103^ However, neither study had examined improvements in exercise capacity.

The beneficial cardiovascular effects of sodium-dependent glucose linked transporter-2 inhibitor (SGLT2i) therapy are well established. In the largest to date SGLT2i trial, DECLARE-TIMI 58, dapagliflozin reduced the risk of death or hospitalization for HF by 17% even in lower-risk patients with T2D.^105^ SGLT2is exert cardioprotective effects which may be beneficial to improving exercise tolerance, including favourable changes in LV mass and wall stress, lowering arterial stiffness and improvements in myocardial energetics.^106^ To our knowledge, only one study to date has examined the effects of SGLT2i on physical function.^104^ In a randomized, double blinded study of dapagliflozin and exercise *versus* dapagliflozin and placebo, the dapagliflozin treatment resulted in 15% increase in exercise capacity from baseline compared with exercise and placebo (VO_2peak_ 2.58 ± 0.63 mL/kg per min *versus* 2.98 ± 0.63 mL/kg per min *p* < 0.001).^104^ The precise mechanisms behind this effect of dapagliflozin are unclear. The proposed mechanisms include improved vascular function,^107^ decreased arterial stiffness,^107^ preferential shift to fatty acid oxidation and ketotic metabolism which is favourable to cardiac energetics,^108^ and weight loss.^107^ These must be interpreted with caution as none of the studies used HF as an end point, nor assessed the role of SGLT2i in improving exercise capacity on a wider scale. Nevertheless, the finding that SGLT2i may improve exercise capacity is of interest, and deserves further examination in larger studies.

## Conclusion

The scale of T2D prevalence has now reached pandemic proportions. Individuals with T2D are at high risk of cardiovascular mortality and HF. It is widely accepted that people with T2D have a baseline reduction in exercise capacity, which confers increased clinical risk of morbidity and mortality. Exercise intolerance can be present in otherwise asymptomatic individuals, and may be the first sign of HF. A multitude of factors contribute to reduced exercise capacity and HF risk in T2D, including metabolic dysregulation, chronic inflammation, endothelial dysfunction, frailty, cardiac systolic and diastolic dysfunction, impaired myocardial energetics, steatosis, calcium homeostasis, coronary microvascular dysfunction and LA myopathy. Whilst there are a number of clinical scoring systems designed to stratify the risk of development of cardiovascular complications in T2D, none of these have been validated for predicting the reduced exercise tolerance in T2D, and thus helping to identify those at risk of HF. Strategies to improve cardiovascular fitness based on combination of diet and exercise appear to be the most efficacious way towards improving outcomes in those with T2D, although newer glucose-lowering therapies may play a key role in preventing HF development in the future.

## References

[1] Savarese G Lund LH Global public health burden of heart failure. Card Fail Rev 2017; 3: 7. 28785469 10.15420/cfr.2016:25:2 PMC5494150

[2] Lloyd-Jones DM Larson MG Leip EP, et al Lifetime risk for developing congestive heart failure: the Framingham Heart Study. Circulation. 2002; 106: 3068–3072. 12473553 10.1161/01.cir.0000039105.49749.6f

[3] Gulsin GS Athithan L McCann GP Diabetic cardiomyopathy: prevalence, determinants and potential treatments. Ther Adv Endocrinol Metab 2019; 10. 10.1177/2042018819834869 PMC6437329 30944723

[4] Facts & Figures - Diabetes UK. https://www.diabetes.org.uk/professionals/position-statements-reports/statistics?gclid=Cj0KCQjw3Nv3BRC8ARIsAPh8hgL4xAM6jnaOYrAZ7PN2sDpBUFJhB5q-m2wKaeXTH27RDdEY_QKsJsIaAp-REALw_wcB (2020, accessed 28 June 2020).

[5] McMurray JJV Packer M Desai AS, et al Angiotensin–neprilysin inhibition versus enalapril in heart failure. N Engl J Med 2014; 371: 993–1004. 25176015 10.1056/NEJMoa1409077

[6] Zareini B Blanche P D’Souza M, et al Type 2 diabetes mellitus and impact of heart failure on prognosis compared to other cardiovascular diseases: a nationwide study. Circ Cardiovasc Qual Outcomes 2020; 13: e006260. 32571092 10.1161/CIRCOUTCOMES.119.006260

[7] MacDonald MR Petrie MC Varyani F, et al Impact of diabetes on outcomes in patients with low and preserved ejection fraction heart failure: an analysis of the Candesartan in Heart failure: assessment of Reduction in Mortality and morbidity (CHARM) programme. Eur Heart J 2008; 29: 1377–1385. 18413309 10.1093/eurheartj/ehn153

[8] Shah AD Langenberg C Rapsomaniki E, et al Type 2 diabetes and incidence of cardiovascular diseases: a cohort study in 1.9 million people. Lancet Diabetes Endocrinol 2015; 3: 105–113. 25466521 10.1016/S2213-8587(14)70219-0 PMC4303913

[9] National Diabetes Audit -2012-2013, Report 2 - NHS Digital. Accessed July 22, 2020 https://digital.nhs.uk/data-and-information/publications/statistical/national-diabetes-audit/national-diabetes-audit-2012-2013-report-2

[10] Reusch JEB Bridenstine M Regensteiner JG Type 2 diabetes mellitus and exercise impairment. Rev Endocr Metab Disord 2013; 14: 77–86. 23299658 10.1007/s11154-012-9234-4 PMC3593997

[11] Bauer TA Reusch JEB Levi M, et al Skeletal muscle deoxygenation after the onset of moderate exercise suggests slowed microvascular blood flow kinetics in type 2 diabetes. Diabetes Care 2007; 30: 2880–2885. 17675540 10.2337/dc07-0843

[12] Nojima H Yoneda M Watanabe H, et al Association between aerobic capacity and the improvement in glycemic control after the exercise training in type 2 diabetes. Diabetol Metab Syndr 2017; 9: 63. 28828040 10.1186/s13098-017-0262-9 PMC5563031

[13] Khan H Kunutsor S Rauramaa R, et al Cardiorespiratory fitness and risk of heart failure: a population-based follow-up study. Eur J Heart Fail 2014; 16: 180–188. 24464981 10.1111/ejhf.37

[14] Ribisl PM Lang W Jaramillo SA, et al Exercise capacity and cardiovascular/metabolic characteristics of overweight and obese individuals with type 2 diabetes: the look AHEAD clinical trial. Diabetes Care 2007; 30: 2679–2684. 17644623 10.2337/dc06-2487

[15] Sacre JW Jellis CL Haluska BA, et al Association of exercise intolerance in type 2 diabetes with skeletal muscle blood flow reserve. JACC Cardiovasc Imaging 2015; 8: 913–921. 26189114 10.1016/j.jcmg.2014.12.033

[16] Avogaro A Albiero M Menegazzo L, et al Endothelial dysfunction in diabetes: the role of reparatory mechanisms. Diabetes Care 2011; 34(Suppl. 2): S285–S290. doi:10.2337/dc11-s239 21525470 PMC3632194

[17] Womack L Peters D Barrett EJ, et al Abnormal skeletal muscle capillary recruitment during exercise in patients with type 2 diabetes mellitus and microvascular complications. J Am Coll Cardiol 2009; 53: 2175–2183. 19497445 10.1016/j.jacc.2009.02.042 PMC2722783

[18] Santoro C Sorrentino R Esposito R, et al Cardiopulmonary exercise testing and echocardiographic exam: an useful interaction. Cardiovasc Ultrasound 2019; 17: 29. 31796047 10.1186/s12947-019-0180-0 PMC6892222

[19] Del Buono G Arena R Borlaug BA, et al Exercise tolerance in patients with heart failure. JACC State of the Art Review. J Am Coll Cardiol 2019; 73: 2210–2225 10.1016/j.jacc.2019.01.072 31047010

[20] Saltiel AR Olefsky JM Inflammatory mechanisms linking obesity and metabolic disease. J Clin Invest 2017; 127: 1–4. 28045402 10.1172/JCI92035 PMC5199709

[21] Shoelson SE Herrero L Naaz A Obesity, inflammation, and insulin resistance. Gastroenterology 2007; 132: 2169–2180. 17498510 10.1053/j.gastro.2007.03.059

[22] Tsalamandris S Antonopoulos AS Oikonomou E, et al The role of inflammation in diabetes: current concepts and future perspectives. Euro Cardiol Rev 2019; 14: 50–59. 10.15420/ecr.2018.33.1 PMC6523054 31131037

[23] Calle MC Fernandez ML Inflammation and type 2 diabetes. Diabetes and Metab 2012; 38: 183–191. 10.1016/j.diabet.2011.11.006 22252015

[24] Lundberg M Seiron P Ingvast S, et al Insulitis in human diabetes: a histological evaluation of donor pancreases. Diabetologia 2017; 60: 346–353. 27796420 10.1007/s00125-016-4140-z PMC6518093

[25] Shi H Kokoeva MV Inouye K, et al TLR4 links innate immunity and fatty acid–induced insulin resistance. J Clin Invest 2006; 116: 3015–3025. 17053832 10.1172/JCI28898 PMC1616196

[26] Hopps E Canino B Caimi G Effects of exercise on inflammation markers in type 2 diabetic subjects. Acta Diabetol 2011; 48: 183–189. 21431832 10.1007/s00592-011-0278-9

[27] Eder K Baffy N Falus A, et al The major inflammatory mediator interleukin-6 and obesity. Inflam Res 2009; 58: 727–736. 10.1007/s00011-009-0060-4 19543691

[28] Schmidt MI Duncan BB Sharrett AR, et al Markers of inflammation and prediction of diabetes mellitus in adults (Atherosclerosis Risk in Communities study): a cohort study. Lancet 1999; 353: 1649–1652. 10335783 10.1016/s0140-6736(99)01046-6

[29] Vepsäläinen T Soinio M Marniemi J, et al Physical activity, high-sensitivity C-reactive protein, and total and cardiovascular disease mortality in type 2 diabetes. Diabetes Care. 2011; 34: 1492–1496. 21602429 10.2337/dc11-0469 PMC3120189

[30] Visser M Bouter LM McQuillan GM, et al Elevated C-reactive protein levels in overweight and obese adults. JAMA 1999; 282: 2131–2135. 10591334 10.1001/jama.282.22.2131

[31] Uysal KT Wiesbrock SM Marino MW, et al Protection from obesity-induced insulin resistance in mice lacking TNF-α function. Nature 1997; 389: 610–614. 9335502 10.1038/39335

[32] Melo LC Dativo-Medeiros J Menezes-Silva CE, et al Physical exercise on inflammatory markers in type 2 diabetes patients: a systematic review of randomized controlled trials. Oxid Med Cell Longev 2017; 2017: 1–10. 10.1155/2017/8523728 PMC5376457 28400914

[33] Kawata T Daimon M Miyazaki S, et al Coronary microvascular function is independently associated with left ventricular filling pressure in patients with type 2 diabetes mellitus. Cardiovasc Diabetol 2015; 14: 98. 26242308 10.1186/s12933-015-0263-7 PMC4525728

[34] Shivu GN Phan TT Abozguia K, et al Relationship between coronary microvascular dysfunction and cardiac energetics impairment in type 1 diabetes mellitus. Circulation. 2010; 121: 1209–1215. 20194884 10.1161/CIRCULATIONAHA.109.873273

[35] Sara JD Taher R Kolluri N, et al Coronary microvascular dysfunction is associated with poor glycemic control amongst female diabetics with chest pain and non-obstructive coronary artery disease. Cardiovasc Diabetol 2019; 18. 30819191 10.1186/s12933-019-0833-1 PMC6393964

[36] Potier L Chequer R Roussel R, et al Relationship between cardiac microvascular dysfunction measured with 82Rubidium-PET and albuminuria in patients with diabetes mellitus. Cardiovasc Diabetol 2018; 17: 11. 29325551 10.1186/s12933-017-0652-1 PMC5763541

[37] Bers DM Eisner DA Valdivia HH Sarcoplasmic reticulum Ca^2+^ and heart failure. Circ Res 2003; 93: 487–490. 14500331 10.1161/01.RES.0000091871.54907.6B

[38] Costa Melo L Dativo-Medeiro J Menzes-Silva CE, et al Physical exercise on inflammatory markers in type 2 diabetes patients: a systemic review of randomized controlled trials. Oxid Med Cell Longev 2017; 8523728. 28400914 10.1155/2017/8523728 PMC5376457

[39] Sidossis L Kajimura S Brown and beige fat in humans: thermogenic adipocytes that control energy and glucose homeostasis. J Clin Invest 2015; 125: 478–486. 25642708 10.1172/JCI78362 PMC4319444

[40] Sugita Y Ito K Sakurai S, et al Epicardial adipose tissue is tightly associated with exercise intolerance in patients with type 2 diabetes mellitus with asymptomatic left ventricular structural and functional abnormalities. J Diabetes Complications 2020; 34. 10.1016/j.jdiacomp.2020.107552 32139127

[41] Babyak MA What you see may not be what you get: a brief, nontechnical introduction to overfitting in regression-type models. Psychosom Med 2004; 66: 411–421. 15184705 10.1097/01.psy.0000127692.23278.a9

[42] Vergès B Patois-Vergès B Iliou MC, et al Influence of glycemic control on gain in VO_2_ peak, in patients with type 2 diabetes enrolled in cardiac rehabilitation after an acute coronary syndrome. The prospective DARE study. BMC Cardiovasc Disord 2015; 15. 26152221 10.1186/s12872-015-0055-8 PMC4495681

[43] Kielstein JT Impraim B Simmel S, et al Cardiovascular effects of systemic nitric oxide synthase inhibition with asymmetrical dimethylarginine in humans. Circulation 2004; 109: 172–177. 14662708 10.1161/01.CIR.0000105764.22626.B1

[44] Fang ZY Sharman J Prins JB, et al Determinants of exercise capacity in patients with type 2 diabetes. Diabetes Care 2005; 28: 1643–1648. 15983314 10.2337/diacare.28.7.1643

[45] Roberts TJ Burns AT MacIsaac RJ, et al Exercise capacity in diabetes mellitus is predicted by activity status and cardiac size rather than cardiac function: a case control study. Cardiovasc Diabetol 2018; 17: 44. 29571290 10.1186/s12933-018-0688-x PMC5866526

[46] Byrkjeland R Edvardsen E Njerve IU, et al Insulin levels and HOMA index are associated with exercise capacity in patients with type 2 diabetes and coronary artery disease. Diabetol Metab Syndr 2014; 6. 24612649 10.1186/1758-5996-6-36 PMC3984726

[47] Gulsin G Henson J Brady E, et al Cardiovascular determinants of aerobic exercise capacity in adults with type 2 diabetes. Diabetes Care 2020; 43: 2248–2256. 32680830 10.2337/dc20-0706 PMC7440912

[48] Vukomanovic V Suzic-Lazic J Celic V, et al The relationship between functional capacity and left ventricular strain in patients with uncomplicated type 2 diabetes. J Hypertens 2019; 37: 1871–1876. 31045966 10.1097/HJH.0000000000002125

[49] Kosmala W

[50] Regensteiner JG Bauer TA Reusch JEB, et al Cardiac dysfunction during exercise in uncomplicated type 2 diabetes. Med Sci Sports Exerc 2009; 41: 977–984. 19346991 10.1249/MSS.0b013e3181942051 PMC2903427

[51] Moser O Eckstein ML McCarthy O, et al Poor glycaemic control is associated with reduced exercise performance and oxygen economy during cardio-pulmonary exercise testing in people with type 1 diabetes. Diabetol Metab Syndr 2017; 9: 93. 29201153 10.1186/s13098-017-0294-1 PMC5697085

[52] Solomon SD McMurray JJV Anand IS, et al Angiotensin–neprilysin inhibition in heart failure with preserved ejection fraction. N Engl J Med 2019; 381: 1609–1620. 31475794 10.1056/NEJMoa1908655

[53] Seok WP Goodpaster BH Jung SL, et al Excessive loss of skeletal muscle mass in older adults with type 2 diabetes. Diabetes Care 2009; 32: 1993–1997. 19549734 10.2337/dc09-0264 PMC2768193

[54] Hanlon P Nicholl BI Jani BD, et al Frailty and pre-frailty in middle-aged and older adults and its association with multimorbidity and mortality: a prospective analysis of 493 737 UK Biobank participants. Lancet Public Health 2018; 3: e323-e332. 29908859 10.1016/S2468-2667(18)30091-4 PMC6028743

[55] Maisch B Alter P Pankuweit S Diabetic cardiomyopathy – fact or fiction? Herz 2011; 36: 102–115. 21424347 10.1007/s00059-011-3429-4

[56] Ponikowski P Voors AA Anker SD, et al 2016 ESC guidelines for the diagnosis and treatment of acute and chronic heart failure. Euro Heart J 2016; 37: 2129–2200. 10.1093/eurheartj/ehw128 27206819

[57] Korosoglou G Humpert PM Ahrens J, et al Left ventricular diastolic function in type 2 diabetes mellitus is associated with myocardial triglyceride content but not with impaired myocardial perfusion reserve. J Magn Reson Imaging 2012; 35: 804–811. 22068959 10.1002/jmri.22879

[58] Jonker JT Djaberi R van Schinkel LD, et al Very-low-calorie diet increases myocardial triglyceride content and decreases diastolic left ventricular function in type 2 diabetes with cardiac complications. Diabetes Care 2014; 37: e1–e2. 24356600 10.2337/dc13-1423

[59] Rijzewijk LJ van der Meer RW Smit JWA, et al Myocardial steatosis is an independent predictor of diastolic dysfunction in type 2 diabetes mellitus. J Am Coll Cardiol 2008; 52: 1793–1799. 19022158 10.1016/j.jacc.2008.07.062

[60] Levelt E Rodgers CT Clarke WT, et al Cardiac energetics, oxygenation, and perfusion during increased workload in patients with type 2 diabetes mellitus. Euro Heart J 2016; 37: 3461–3469. 10.1093/eurheartj/ehv442 PMC5201143 26392437

[61] Greenberg B Butler J Felker GM, et al Calcium upregulation by percutaneous administration of gene therapy in patients with cardiac disease (CUPID 2): a randomised, multinational, double-blind, placebo-controlled, phase 2b trial. Lancet 2016; 387: 1178–1186. 26803443 10.1016/S0140-6736(16)00082-9

[62] Gianni D Chan J Gwathmey JK, et al SERCA2a in heart failure: role and therapeutic prospects. J Bioenerg Biomembr 2005; 37: 375–380. 16691468 10.1007/s10863-005-9474-z

[63] Pereira L Ruiz-Hurtado G Rueda A, et al Calcium signaling in diabetic cardiomyocytes. Cell Calcium 2014; 56: 372–380. 25205537 10.1016/j.ceca.2014.08.004

[64] Jaski BE Jessup ML Mancini DM, et al Calcium upregulation by percutaneous administration of gene therapy in cardiac disease (CUPID Trial), a first-in-human phase 1/2 clinical trial. J Card Fail 2009; 15: 171–181. 19327618 10.1016/j.cardfail.2009.01.013 PMC2752875

[65] Boonman-De Winter LJM Rutten FH Cramer MJM, et al High prevalence of previously unknown heart failure and left ventricular dysfunction in patients with type 2 diabetes. Diabetologia 2012; 55: 2154–2162. 22618812 10.1007/s00125-012-2579-0 PMC3390708

[66] Kanagala P Arnold JR Singh A, et al Characterizing heart failure with preserved and reduced ejection fraction: an imaging and plasma biomarker approach. PLoS One 2020; 15: e0232280. 32349122 10.1371/journal.pone.0232280 PMC7190371

[67] Mazumder PK O’Neill BT Roberts MW, et al Impaired cardiac efficiency and increased fatty acid oxidation in insulin-resistant ob/ob mouse hearts. Diabetes 2004; 53: 2366–2374. 15331547 10.2337/diabetes.53.9.2366

[68] Athithan L Gulsin GS McCann GP, et al Diabetic cardiomyopathy: pathophysiology, theories and evidence to date. World J Diabetes 2019; 10: 490–510. 31641426 10.4239/wjd.v10.i10.490 PMC6801309

[69] Skali H Shah A Gupta DK, et al Cardiac structure and function across the glycemic spectrum in elderly men and women free of prevalent heart disease: the atherosclerosis risk in the community study. Circ Heart Fail 2015; 8: 448–454. 25759458 10.1161/CIRCHEARTFAILURE.114.001990 PMC4439326

[70] Ng ACT Delgado V Bertini M, et al Findings from left ventricular strain and strain rate imaging in asymptomatic patients with type 2 diabetes mellitus. Am J Cardiol 2009; 104: 1398–1401. 19892057 10.1016/j.amjcard.2009.06.063

[71] Liu X Yang ZG Gao Y, et al Left ventricular subclinical myocardial dysfunction in uncomplicated type 2 diabetes mellitus is associated with impaired myocardial perfusion: A contrast-enhanced cardiovascular magnetic resonance study. Cardiovasc Diabetol 2018; 17. 10.1186/s12933-018-0782-0 PMC6206833 30373588

[72] Roos CJ Scholte AJ Kharagjitsingh AV, et al Changes in multidirectional LV strain in asymptomatic patients with type 2 diabetes mellitus: a 2-year follow-up study. Eur Heart J Cardiovasc Imaging 2014; 15: 41–47. 23793876 10.1093/ehjci/jet075

[73] Hasselberg NE Haugaa KH Sarvari SI, et al Left ventricular global longitudinal strain is associated with exercise capacity in failing hearts with preserved and reduced ejection fraction. Eur Heart J Cardiovasc Imaging 2015; 16: 217–224. 25552469 10.1093/ehjci/jeu277 PMC4307775

[74] She F Ma Y Li Y, et al Influence of heart rate control on exercise capacity and quality of life in patients with permanent atrial fibrillation. BMC Cardiovasc Disord 2019; 19. 31864289 10.1186/s12872-019-01293-3 PMC6925461

[75] Sørensen MH Bojer AS Pontoppidan JRN, et al Reduced myocardial perfusion reserve in type 2 diabetes is caused by increased perfusion at rest and decreased maximal perfusion during stress. Diabetes Care 2020; 43: 1285–1292. 32193248 10.2337/dc19-2172

[76] Sørensen MH Bojer AS Broadbent DA, et al Cardiac perfusion, structure, and function in type 2 diabetes mellitus with and without diabetic complications. Eur Heart J Cardiovasc Imaging. Epub ahead of print 23 October 2019. DOI:10.1093/ehjci/jez266. 31642902

[77] Steadman CD Jerosch-Herold M Grundy B, et al Determinants and functional significance of myocardial perfusion reserve in severe aortic stenosis. JACC: Cardiovasc Imaging. 2012; 5: 182–189. 22340825 10.1016/j.jcmg.2011.09.022

[78] Jensen MT Fung K Aung N, et al Changes in cardiac morphology and function in individuals with diabetes mellitus: the UK biobank cardiovascular magnetic resonance substudy. Circ Cardiovasc Imaging 2019; 12. 10.1161/CIRCIMAGING.119.009476 PMC7099857 31522551

[79] Ugowe FE Jackson LR Thomas KL Atrial fibrillation and diabetes mellitus: can we modify stroke risk through glycemic control? Circ Arrhythm Electrophysiol 2019; 12: e007351. 30995870 10.1161/CIRCEP.119.007351

[80] Nakade T Shirakura T Murata M Effect of atrial fibrillation on cardiac output, exercise tolerance and heart rate response during exercise. Eur Heart J 2017; 38.27071821

[81] Hohendanner F Messroghli D Bode D, et al Atrial remodelling in heart failure: recent developments and relevance for heart failure with preserved ejection fraction. ESC Heart Fail 2018; 5: 211–221. 10.1002/ehf2.12260 PMC5880666 29457877

[82] Prioli A Marino P Lanzoni L, et al Increasing degrees of left ventricular filling impairment modulate left atrial function in humans. Am J Cardiol 1998; 82: 756–761. 9761086 10.1016/s0002-9149(98)00452-4

[83] von Roeder M Rommel K-P Kowallick JT, et al Influence of left atrial function on exercise capacity and left ventricular function in patients with heart failure and preserved ejection fraction. Circ Cardiovasc Imaging 2017; 10. 10.1161/CIRCIMAGING.116.005467 28360259

[84] Kusunose K Motoki H Popovic ZB, et al Independent association of left atrial function with exercise capacity in patients with preserved ejection fraction. Heart. 2012; 98: 1311–1317. 22760870 10.1136/heartjnl-2012-302007

[85] Ratanasit N Karaketklang K Chirakarnjanakorn S, et al Left atrial volume as an independent predictor of exercise capacity in patients with isolated diastolic dysfunction presented with exertional dyspnea. Cardiovasc Ultrasound 2014; 12: 19. 24929939 10.1186/1476-7120-12-19 PMC4074581

[86] Tsang TSM Barnes ME Gersh BJ, et al Left atrial volume as a morphophysiologic expression of left ventricular diastolic dysfunction and relation to cardiovascular risk burden. Am J Cardiol 2002; 90: 1284–1289. 12480035 10.1016/s0002-9149(02)02864-3

[87] Hoit BD Left atrial size and function: Role in prognosis. J Am Col Cardiol 2014; 63: 493–505. 10.1016/j.jacc.2013.10.055 24291276

[88] Tan YT Wenzelburger F Lee E, et al Reduced left atrial function on exercise in patients with heart failure and normal ejection fraction. Heart. 2010; 96: 1017–1023. 20584857 10.1136/hrt.2009.189118

[89] Kadappu KK Boyd A Eshoo S, et al Changes in left atrial volume in diabetes mellitus: more than diastolic dysfunction? Euro Heart J Cardiovasc Imaging 2012; 13: 1016–1023. 10.1093/ehjci/jes084 22544873

[90] Poulsen MK Dahl JS Henriksen JE, et al Left atrial volume index: relation to long-term clinical outcome in type 2 diabetes. J Am Coll Cardiol 2013; 62: 2416–2421. 24076532 10.1016/j.jacc.2013.08.1622

[91] Melenovsky V Hwang SJ Redfield MM, et al Left atrial remodeling and function in advanced heart failure with preserved or reduced ejection fraction. Circ Heart Fail 2015; 8: 295–303. 25593126 10.1161/CIRCHEARTFAILURE.114.001667

[92] Santos ABS Kraigher-Krainer E Gupta DK, et al Impaired left atrial function in heart failure with preserved ejection fraction. Euro J Heart Fail 2014; 16: 1096–1103. 10.1002/ejhf.147 PMC5535768 25138249

[93] Ha J-W Oh JK Pellikka PA, et al Diastolic stress echocardiography: a novel noninvasive diagnostic test for diastolic dysfunction using supine bicycle exercise Doppler echocardiography. J Am Soc Echocardiogr 2005; 18: 63–68. 15637491 10.1016/j.echo.2004.08.033

[94] Arruda ALM Pellikka PA Olson TP, et al Exercise capacity, breathing pattern, and gas exchange during exercise for patients with isolated diastolic dysfunction. J Am Soc Echocardiogr 2007; 20: 838–846. 17617310 10.1016/j.echo.2006.12.006

[95] Skaluba SJ Litwin SE Mechanisms of exercise intolerance: insights from tissue Doppler imaging. Circulation 2004; 109: 972–977. 14967722 10.1161/01.CIR.0000117405.74491.D2

[96] Grewal J McCully RB Kane GC, et al Left ventricular function and exercise capacity. JAMA 2009; 301: 286–294. 19155455 10.1001/jama.2008.1022 PMC2862454

[97] Rider OJ Francis JM Ali MK, et al Beneficial cardiovascular effects of bariatric surgical and dietary weight loss in obesity. J Am Coll Cardiol 2009; 54: 718–726. 19679250 10.1016/j.jacc.2009.02.086

[98] Rider OJ Francis JM Tyler D, et al Effects of weight loss on myocardial energetics and diastolic function in obesity. Int J Cardiovasc Imaging 2013; 29: 1043–1050. 23269470 10.1007/s10554-012-0174-6

[99] Weiss EP Racette SB Villareal DT, et al Lower extremity muscle size and strength and aerobic capacity decrease with caloric restriction but not with exercise-induced weight loss. J Appl Physiol 2007; 102: 634–640. 17095635 10.1152/japplphysiol.00853.2006 PMC4376253

[100] Villareal DT Chode S Parimi N, et al Weight loss, exercise, or both and physical function in obese older adults. N Engl J Med 2011; 364: 1218–1229. 21449785 10.1056/NEJMoa1008234 PMC3114602

[101] Weiss EP Jordan RC Frese EM, et al Effects of weight loss on lean mass, strength, bone, and aerobic capacity. Med Sci Sports Exerc 2017; 49: 206–217. 27580151 10.1249/MSS.0000000000001074 PMC5161655

[102] Gulsin GS Swarbrick DJ Athithan L, et al Effects of low-energy diet or exercise on cardiovascular function in working-age adults with type 2 diabetes: a prospective, randomized, open-label, blinded end point trial. Diabetes Care 2020; 43: 1300–1310. 32220917 10.2337/dc20-0129

[103] Husain M Birkenfeld AL Donsmark M, et al Oral semaglutide and cardiovascular outcomes in patients with type 2 diabetes. N Engl J Med 2019; 381: 841–851. 31185157 10.1056/NEJMoa1901118

[104] Newman AA Grimm NC Wilburn JR, et al Influence of sodium glucose cotransporter 2 inhibition on physiological adaptation to endurance exercise training. J Clin Endocrinol Metab 2019; 104: 1953–1966. 30597042 10.1210/jc.2018-01741

[105] Lee DM Battson ML Jarrell DK, et al SGLT2 inhibition via dapagliflozin improves generalized vascular dysfunction and alters the gut microbiota in type 2 diabetic mice. Cardiovasc Diabetol 2018; 17. 29703207 10.1186/s12933-018-0708-x PMC5921754

[106] Bekki M Tahara N Tahara A, et al Switching dipeptidyl peptidase-4 inhibitors to tofogliflozin, a selective inhibitor of sodium-glucose cotransporter 2 improve arterial stiffness evaluated by cardio-ankle vascular index in patients with type 2 diabetes: a pilot study. Curr Vasc Pharmacol 2019; 17: 411–420. 29766812 10.2174/1570161116666180515154555

[107] Ji L Ma J Li H, et al Dapagliflozin as monotherapy in drug-naive Asian patients with type 2 diabetes mellitus: a randomized, blinded, prospective phase III study. Clin Therap 2014; 36. 10.1016/j.clinthera.2013.11.002 24378206

[108] Ferrannini E Baldi S Frascerra S, et al Shift to fatty substrate utilization in response to sodium-glucose cotransporter 2 inhibition in subjects without diabetes and patients with type 2 diabetes. Diabetes. 2016; 65: 1190–1196. 26861783 10.2337/db15-1356

